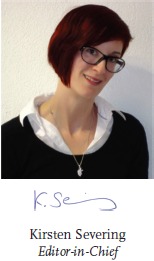# A Successful First Year

**DOI:** 10.1002/advs.201500409

**Published:** 2016-01-13

**Authors:** Kirsten Severing

When we launched *Advanced Science* approximately one year ago, we did it with the intention to establish a publishing platform for your top research in a premium open access environment. 13 months after the launch, we can state that this goal has been reached.

At the time of writing (early December 2015) we have published 11 issues with a total of 107 papers. These papers have already collected a respectable sum of 140 citations. The most cited papers so far are listed in Table 1. The top two papers both come from members of the executive advisory board: Frederik Krebs (Denmark) and Yanglong Hou (China). Also, other board members have contributed great papers during the first year, including Dan Wang (China), Thomas Anthopoulos (UK), Geoffrey Ozin (Canada), Zhenan Bao (USA), Minghua Liu (China), and Frank Caruso (Australia). But it is not only board members who have discovered that *Advanced Science* is a promising platform for their work. We have been delighted to publish research by Richard Friend and Henning Sirringhaus (Cambridge, UK), Andrey Rogach (Hong Kong, China), Krzysztof Matyjaszewski (Pittsburgh, USA), Alex Jen (Seattle, USA), Xavier Crispin (Norrköping, Sweden), and Bin Liu (Singapore).

**Table 1:** Most cited papers in Advanced Science.

Title	Authors	Origin	Published online	DOI	Citations
High‐Volume Processed, ITO‐Free Superstrates and Substrates for Roll‐to‐Roll Development of Organic Electronics	Markus Hösel, Dechan Angmo, Roar R. Søndergaard, Gisele A. dos Reis Benatto, Jon E. Carlé, Mikkel Jørgensen, Frederik C. Krebs	Technical University of Denmark, Roskilde, Denmark	22 November 2014	10.1002/advs.201400002	12
Electrode Nanostructures in Lithium‐Based Batteries	Nasir Mahmood, Yanglong Hou	Peking University, Beijing, China	29 December 2014	10.1002/advs.201400012	10
Phosphorus‐Graphene Nanosheet Hybrids as Lithium‐Ion Anode with Exceptional High‐Temperature Cycling Stability	Zhaoxin Yu, Jiangxuan Song, Mikhail L. Gordin, Ran Yi, Duihai Tang, Donghai Wang	Pennsylvania State University, University Park, PA, USA	28 January 2015	10.1002/advs.201400020	9
Growth of Ultrathin ZnCo_2_O_4_ Nanosheets on Reduced Graphene Oxide with Enhanced Lithium Storage Properties	Guoxin Gao, Hao Bin Wu, Bitao Dong, Shujiang Ding, Xiong Wen (David) Lou	Nanyang Technological University, Singapore	21 January 2015	10.1002/advs.201400014	9
Nanoparticle–Hydrogel Composites: Concept, Design, and Applications of These Promising, Multi‐Functional Materials	Praveen Thoniyot, Mein Jin Tan, Anis Abdul Karim, David James Young, Xian Jun Loh	National University of Singapore, Singapore	21 January 2015	10.1002/advs.201400010	8

While the majority of contributions published in *Advanced Science* so far focusses on topics that could also be covered in *Advanced Materials* or its sister journals, we are gradually moving into a broader topical spectrum. Papers on improved or combined processes to produce materials can be found next to research on cardiac tissue repair and triple‐negative breast cancer.

**Figure 1:**
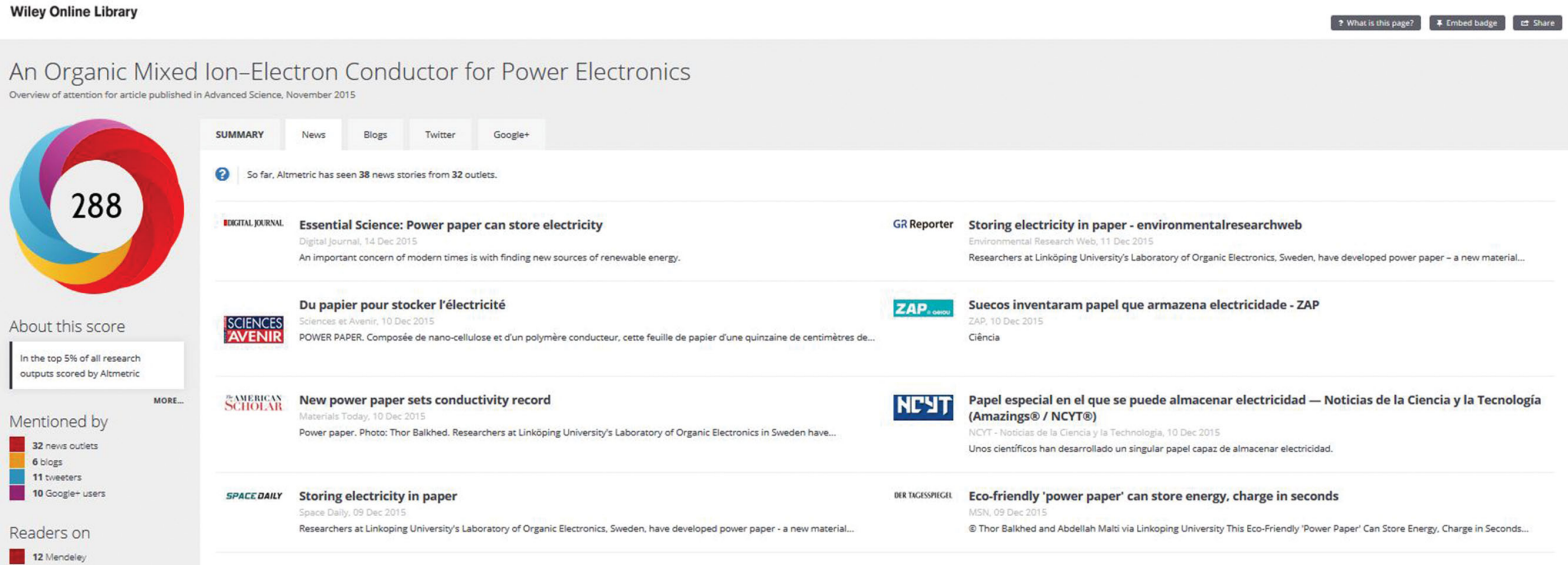
Media reports on article 10.1002/advs.201500305 by X. Crispin and co‐workers.

What's more, *Advanced Science* is already well recognized by the community. The number of submitted manuscripts exceeded our expectations for the first year, and thus, hard choices had to be made. Several papers that did not make it into *Advanced Science* could be transferred into more specialized titles in the *Advanced Materials* family, indicating that the readers accept the superior position intended for *Advanced Science*. Also, the number of full text downloads of papers published in *Advanced Science* shows a very positive trend reflecting the high interest in the journal. Several of the papers published in the journal were picked up by the media; an example can be seen in Figure 1. This research by Swedish researchers Xavier Crispin, Magnus Berggren, and co‐workers even made it onto the German radio (Deutschlandfunk). Yen‐Hung Lin (from Thomas Anthopoulos’ group at Imperial College, London, UK) won the Graduate Student Award at the MRS Fall Meeting in Boston for his *Advanced Science* paper on low‐dimensional semiconductors for next ­generation large area electronics. More than 100 researchers joined our reception in Boston (including Thomas Anthopoulos, Siegfried Bauer, Ali Khademhosseini, and Fei Wei—all members of the executive advisory board) to celebrate the first successful year of *Advanced Science* and the achievements of the whole *Advanced Materials* family.

We are determined to continue our ­successful journey in 2016 and wish you all a Happy New Year!